# Fungal Keratitis, or Misled by a Small Insect?

**DOI:** 10.4274/tjo.galenos.2019.30670

**Published:** 2020-04-29

**Authors:** Betül Nurefşan Bayraktutar, Ayse Yıldız Taş, Afsun Şahin

**Affiliations:** 1 Cornea Service, New England Eye Center, Department of Ophthalmology, Tufts Medical School, Tufts University School of Medicine, Boston, USA; 2 Koç University Hospital, Department of Ophtalmology, İstanbul, Turkey; 3 Koç University Faculty of Medicine, Department of Ophtalmology, İstanbul, Turkey

**Keywords:** Pine processionary caterpillar, fungal keratitis, in vivo confocal microscopy, keratitis ophthalmia nodosa

## Abstract

The pine processionary caterpillar is an insect that has multiple small, thin hairs around its body as a defense mechanism. These hairs have a hazardous effect on ocular structures and cause a broad range of reactions from conjunctivitis to endophthalmitis, referred to as ophthalmia nodosa. The diagnosis of the disease is based on the patient’s history and the detection of the hairs on ocular structures. In this report, we present a patient with ophthalmia nodosa misdiagnosed as fungal keratitis, and the actual diagnosis was made by *in vivo* confocal microscopy. We also would like to increase awareness among ophthalmologists about the disease which has a growing distribution area due to climate change.

## Introduction

The pine processionary caterpillar, *Thaumetopoea pityocampa*, is an insect which has abundant fine hairs (also called setae) on its body. It is widely distributed in the pine forests of warmer regions in southern Europe, central Asia, the Near East, and North Africa.^1^

The hairs of the pine processionary caterpillar have hazardous effects on the human eye, skin, and respiratory tract. The clinical presentation of the resulting ocular disease, referred to as ophthalmia nodosa, can vary in a wide spectrum as conjunctivitis, keratitis, cataracts, uveitis, vitritis, and endophthalmitis.^2,3,4,5,6,7,8^ The ocular reaction is linked to the mechanical effect of the hairs, direct toxicity of the toxin present inside the hair, and immunoglobulin E-mediated allergic reaction to various caterpillar proteins. The diagnosis of the disease is based on direct visualization of hairs and clinical history of the patient. Since it is a rare condition and the hairs are extremely small and thin, it can be mis- or underdiagnosed during a routine slit-lamp examination.

Our aim in this case report is to present a patient who was misdiagnosed as having fungal keratitis and increase the awareness of ophthalmologists about ophthalmia nodosa, which is expected to be seen at higher frequency with a wider distribution area^1^ because of global warming.

## Case Report

A 74-year-old man was referred to our clinic with a presumed fungal keratitis diagnosis from a general ophthalmologist. The patient presented to the other clinic with the complaint of acute-onset eyelid swelling, redness, pain, and vision loss in his right eye. He was diagnosed with presumed fungal keratitis and treated with topical fortified antibiotic and antifungal therapy. Corneal scraping and culture was reported to be negative. However, his clinical signs and symptoms worsened despite topical antibacterial and antifungal therapy, and he was referred to our clinic for a definitive diagnosis.

**Figure 1:**
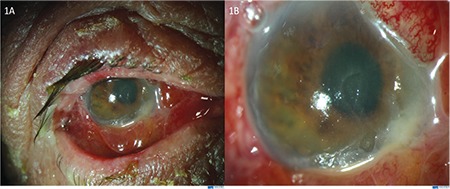
**A)** Severe eyelid edema, conjunctival hyperemia, and chemosis are seen in the right eye. **B)** Diffuse corneal edema, multiple foci of keratitis, fibrinous anterior chamber reaction, and 3-mm asymmetric hypopyon in the right eye are seen in the right eye

**Figure 2:**
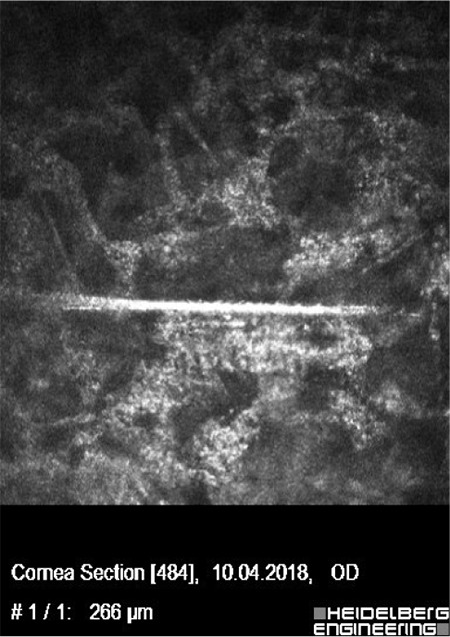
*In vivo* confocal microscopic image of caterpillar hair: Linear needle-shaped structure with small protrusions, which resembles fungal hyphae

**Figure 3:**
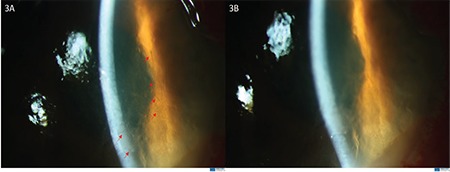
**A)** Numerous caterpillar hair (red arrows) embedded in cornea were visualized in slit-lamp examination using retroillumination from the iris. **B)** Corneal appearance after corneal debridement was performed under a surgical operating microscope

On examination in our clinic, the right periocular skin including the upper and lower lids of the right eye was severely hyperemic, swollen, and itchy (Figure 1A). Visual acuity was hand movements in the right eye, 20/20 in the left eye. Slit-lamp biomicroscopy revealed conjunctival chemosis, corneal epithelial defect, diffuse corneal haze (more dense in various foci), multiple foci of keratitis, fibrinous anterior chamber reaction, and 3-mm asymmetric hypopyon in the right eye (Figure 1B). The fundus could not be visualized. B-scan ultrasound of the posterior segment was normal. In vivo corneal confocal microscopy (IVCM) was performed for prompt diagnosis. Numerous hyperreflective, linear needle-shaped structures with small protrusions, resembling fungal hyphae, were seen (Figure 2). However, the length and sharp linearity of the structures were not consistent with typical hyphae structures seen in IVCM images. Therefore, the patient’s medical history was re-evaluated in order to understand the events leading to keratitis. The patient was asked for the details of organic trauma before his complaints started and it was learned that he had seen a small tent-like nest in a pine tree in the garden of his home and had tried to remove it from the tree. With this new history, the patient was evaluated again by high-magnification slit-lamp examination and numerous caterpillar hairs embedded in the cornea were seen with iris-scattering lighting (Figure 3A). These hairs were not seen at any other structure, including the anterior chamber, iris, vitreous, and retina.

**Figure 4:**
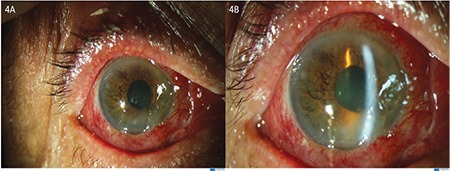
On post-treatment day 3, **A)** eyelid edema was decreased and **B)** conjunctival chemosis and hypopyon disappeared, corneal edema started to resolve

**Figure 5:**
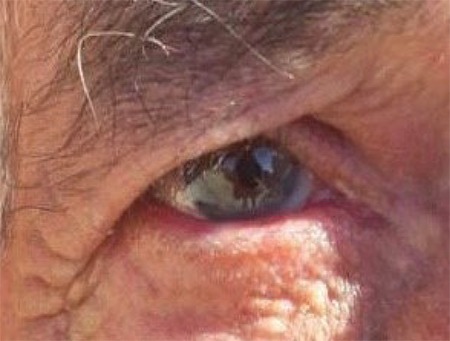
Photograph of the patient at post-treatment 1 month. Eyelid and anterior segment were totally quiet

Corneal debridement was performed under a surgical operating microscope (Figure 3B). The patient was treated with topical prednisolone acetate 1.0% solution hourly, cycloplegic eye drops 3 times a day, and moxifloxacin 0.5% eye drops 4 times a day as prophylaxis against secondary infection. On the third day of treatment, his visual acuity increased to 20/200, eyelid edema and conjunctival chemosis decreased (Figure 4A), hypopyon disappeared, +1 anterior chamber reaction was seen, and the corneal edema began to resolve (Figure 4B). Steroid therapy was tapered slowly over a month and at 1 month his visual acuity returned to 20/20 and all clinical signs resolved (Figure 5).

## Discussion

In this article, we report a rare condition called ophthalmia nodosa, which was misdiagnosed as fungal keratitis and later differentially diagnosed using IVCM. Although the disease was first described in the late 1800s, it is not generally well known by ophthalmologists.^9^ It is caused by a reaction to the hairs of a certain caterpillar which generally lives in pine forests but can be seen wherever pine trees are present. Therefore, the distribution of the disease includes warmer regions in southern Europe, the Near East, and North Africa with an expectation induced by global warming.^2^

The caterpillar is covered by abundant small, spined urticating hairs as a defense mechanism. When these hairs come into contact with the ocular surface, the clinical presentation may vary in a wide range from allergic conjunctivitis to endophthalmitis, since the hairs can penetrate into the eye.^2,3,4,5,6,8,9,10^ The diagnosis is based on patient history and clinically detected caterpillar hairs. However, because of the small size of the hairs, they cannot be seen in a routine slit-lamp examination and the diagnosis can be missed. In this patient, IVCM was instrumental in visualizing the caterpillar hairs and discriminating the clinical presentation from infectious keratitis. It was crucial for this patient that the treatment regimen was changed immediately from antifungal to steroid therapy.

*In vivo* confocal microscopy is a non-invasive, high-resolution, real-time device which is widely used for the diagnosis and treatment follow-up of many anterior segment diseases such as dry eye, keratitis, corneal dystrophies, post-refractive surgery, and post-keratoplasty. To the best of our knowledge, this is the second case in which IVCM was used as a diagnostic tool for caterpillar hairs, which appear as hyperreflective linear needle shapes with multiple small spines.^11^ The diagnosis of ophthalmia nodosa may be very challenging in areas where these caterpillars are not common, and IVCM might assist with these specific findings.

It is important to be aware of the disease and treat immediately due to the penetration capacity of the hairs. In 2017, an entomology report pointed out that the insect responded to climate changes and insect distribution areas and their natural predators were waxing.^1^ Therefore, in the near future many ophthalmologists may encounter this entity for the first time, which may result in late or misdiagnosis.

In conclusion, ophthalmologists should be aware of ocular conditions associated with caterpillar hairs and keep them in mind in patients with organic traumas, especially when pine tree contact is present. In patients with insignificant history and clinically undetectable hairs, IVCM can be used as a diagnostic tool.

## References

[1] Battisti A, Larsson S, Roques A (2017). Processionary Moths and Associated Urtication Risk: Global Change-Driven Effects. Annu Rev Entomol..

[2] Portero A, Carreno E, Galarreta D, Herreras JM (2013). Corneal inflammation from pine processionary caterpillar hairs. Cornea..

[3] Horng CT, Chou PI, Liang JB (2000). Caterpillar setae in the deep cornea and anterior chamber. Am J Ophthalmol..

[4] Gonzalez-Martin-Moro J, Contreras-Martin I, Castro-Rebollo M, Fuentes- Vega I, Zarallo-Gallardo J (2019). Focal cortical cataract due to caterpillar hair migration. Clin Exp Optom..

[5] Conrath J, Hadjadj E, Balansard B, Ridings B (2000). Caterpillar setae-induced acute anterior uveitis: a case report. Am J Ophthalmol..

[6] Fraser SG, Dowd TC, Bosanquet RC (1994). Intraocular caterpillar hairs (setae): clinical course and management. Eye (Lond)..

[7] Singh R, Tripathy K, Chawla R, Khokhar S (2017). Caterpillar hair in the eye. BMJ Case Rep..

[8] Shibui H, Kawashima H, Kamata K, Sasaki H, Inoda S, Shimizu H (1997). Vitrectomy for caterpillar seta-induced endophthalmitis. Arch Ophthalmol..

[9] Teske SA, Hirst LW, Gibson BH, O’Connor PA, Watts WH, Carey TM (1991). Caterpillar-induced keratitis. Cornea..

[10] Agarwal M, Acharya MC, Majumdar S, Paul L (2017). Managing multiple caterpillar hair in the eye. Indian J Ophthalmol..

[11] Jullienne R, He Z, Manoli P, Grivet D, Cinotti E, Perrot JL, Labeille B, Cambazard F, Gain P, Thuret G (2015). In vivo confocal microscopy of pine processionary caterpillar hair-induced keratitis. Cornea..

